# Environmental/lifestyle factors in the pathogenesis and prevention of type 2 diabetes

**DOI:** 10.1186/s12916-017-0901-x

**Published:** 2017-07-19

**Authors:** Hubert Kolb, Stephan Martin

**Affiliations:** 1 Faculty of Medicine, University of Duesseldorf, Duesseldorf, Germany; 2 West-German Centre of Diabetes and Health, Duesseldorf Catholic Hospital Group, Hohensandweg 37, 40591 Duesseldorf, Germany

**Keywords:** Type 2 diabetes, Environment, Lifestyle, Diabetes risk factors, Diabetes prevention, Diet, Physical activity, β-cells

## Abstract

**Background:**

Environmental and lifestyle changes, in addition to the ageing of populations, are generally believed to account for the rapid global increase in type 2 diabetes prevalence and incidence in recent decades.

**Discussion:**

In this review, we present a comprehensive overview of factors contributing to diabetes risk, including aspects of diet quality and quantity, little physical activity, increased monitor viewing time or sitting in general, exposure to noise or fine dust, short or disturbed sleep, smoking, stress and depression, and a low socioeconomic status. In general, these factors promote an increase in body mass index. Since loss of β-cell function is the ultimate cause of developing overt type 2 diabetes, environmental and lifestyle changes must have resulted in a higher risk of β-cell damage in those at genetic risk. Multiple mechanistic pathways may come into play.

**Conclusions:**

Strategies of diabetes prevention should aim at promoting a ‘diabetes-protective lifestyle’ whilst simultaneously enhancing the resistance of the human organism to pro-diabetic environmental and lifestyle factors. More research on diabetes-protective mechanisms seems warranted.

## Background

Over the past decades, there has been a major increase in type 2 diabetes (T2D) prevalence in most regions of the world [1]. After adjusting for the impact of ageing populations, diabetes prevalence in adults (85–95% T2D) almost doubled between 1980 and 2014 worldwide. Increases were more pronounced in low- and middle-income countries and in men compared to women [1].

Recognition of the environmental and lifestyle factors responsible for these changes in theory may lead to the development of strategies to decrease the number of new cases to reach those of 20–40 years earlier. This review presents the current state of knowledge and discusses the possible mechanisms involved and the consequences for strategies of diabetes prevention.

## Overview of environmental and lifestyle factors increasing T2D risk

It is generally believed that an energy-dense Western style diet in conjunction with a sedentary lifestyle are the primary cause of T2D [2]. These two factors are also held responsible for the current global epidemic of obesity, which is closely associated with the rising rate of T2D [3]. At closer analysis, a high body mass index (BMI) appears to contribute less to an increased risk of T2D than the presence of increased visceral obesity [4] and/or ectopic fat (liver fat) [5–7]. This fits with the observation that obese people without metabolic dysregulation have little visceral obesity or liver fat [8–10]. Conversely, people who develop T2D despite being merely overweight or within a normal weight range, such as in Asia, exhibit visceral obesity and ectopic fat deposition and reduced muscle mass, together resulting in a normal or near normal BMI [11–13]. Interestingly, a substantial age-corrected rise of T2D cases in recent decades was also seen in countries with no major change in the availability of food such as in Western Europe [1]. This suggests that additional environmental and lifestyle factors contributed to the increased risk of T2D. A list of factors associated with risk of T2D is shown in Box 1.

### Diet

When considering the wide range of diet types consumed in different regions of the world, it may not be surprising that prospective epidemiological studies vary somewhat in the association of food groups with incident T2D. In general, plant food is associated with lower T2D risk than meat, low energy density food is considered more protective than high density energy food, associations of fish consumption with diabetes risk are variable, and fermented dairy products may be more beneficial than non-fermented ones. Further, refined grains or sugar-sweetened beverages consistently appear to promote obesity and diabetes risk [14–24]. Daily consumption of a handful of nuts may afford some protection from T2D, despite nuts representing a high energy density food [25]. Unfortunately, epidemiological studies cannot exclude the impact of confounding factors such as those of physical activity, which is difficult to assess in queries or interviews.

The most recent recommendation by the Government of the USA no longer focusses on setting limits for the amount of carbohydrates, fat and protein in foods but rather proposes food types or patterns such as a healthy US-style eating pattern, a Mediterranean diet or a vegetarian diet [26].

In order to test for cause-effect relationships, the effect of a given diet on metabolic control has been studied extensively in controlled trials with the assumption that short-term effects may indicate long-term outcomes. In the results published, most of the diets assessed were reported to improve metabolic control and lipid status, regardless of whether they were high or low in carbohydrates, fats or protein [27–31]. In many of these studies, given the unavoidable study effect (Hawthorne effect) on eating behaviour, participants consumed less calories than before the trial, at least during the first weeks, and therefore lost weight. In other trials, a hypocaloric diet was given to all study groups [31]. As a consequence, weight reduction (and concomitantly less visceral obesity) probably led to improved metabolic control, largely independent of the diet tried. Hence, the outcome of many dietary trials may give rise to misleading conclusions on the suitability of a diet for diabetes prevention in the long term.

Unfortunately, long-term trials of selected food groups for diabetes prevention in persons at risk are difficult to perform due to poor compliance. In one successful attempt, a Mediterranean diet supplemented with either 1 L of virgin olive oil per week or 30 g nuts per day was compared, in a randomised trial, with a conventional low fat diet [32]. After 4 years, both Mediterranean diet groups exhibited a rate of progression to T2D of approximately 50% of the control low fat diet group (PREDIMED trial, group size approximately 140 persons, mean age 67 years, high cardiovascular disease risk). In this trial, calorie restriction was not intended and, on average, there was less than 1 kg of body weight loss per person, despite the major reduction of diabetes risk over 4 years. This suggests that there are components in the Mediterranean diet or the overall pattern that may decrease diabetes risk without the need of weight reduction. Confirmation of these data in a larger trial including also younger persons without high cardiovascular disease risk would offer a robust basis for a diabetes prevention guideline. There have been many trials of lifestyle improvement for the prevention of T2D in persons at increased risk, but these do not offer advice on a ‘diabetes-protective’ diet because dietary guidelines have always been combined with a strategy to reduce body weight [33, 34].

Can we learn from the diet of centenarians? Data for Okinawa, Sicily, Sardinia, Linda Loma (California, USA) and Bama county (China) suggest some similarity to the above conclusions from epidemiological studies of a ‘healthy’ diet, i.e. a dominance of vegetables and fruit over animal food, and of whole grains over refined carbohydrates [35–38]. However, it should not be overlooked that there is a substantial contribution from other factors to healthy ageing, such as genetic background, epigenetic DNA methylation status, physical activity or daily work until high age, an active social network, and low smoking rates and alcohol consumption [39, 40].

### Occupational and leisure time physical activity

Epidemiological studies suggest that high versus low total physical activity is associated with a reduction in relative diabetes risk by approximately 30%. All types of leisure time physical activities as well as occupational physical activity were found to be inversely associated with diabetes risk [41, 42]. The beneficial effect of exercise on insulin sensitivity and glycaemic control (by continuous glucose measurement) has also been demonstrated in controlled trials in non-diabetic individuals [43, 44]. Reallocation of 30 min of sedentary time into moderate to vigorous physical activity was associated with a 15% difference in HOMA-defined insulin sensitivity [45]. The beneficial effects of muscle work do not simply reflect the burning of calories, since enhanced physical activity leads to minimal weight loss [46].

### Watching TV or sedentary time

There is a strong association between sedentary time (self-reported or objectively measured) with obesity or incident diabetes, independent of the extent of physical activity [47–51]. Increased duration of sedentary behaviour may double diabetes risk [47]. In one study, each hour of television watching increased the risk of developing diabetes over 3.2 years by 3.4% [49]. Not surprisingly, the interaction appears to be bidirectional – a sedentary lifestyle promotes obesity and vice versa [52].

Recommendations of limiting sedentary time in favour of being in upright posture and moving are based on short-term trials (reviewed in [53]) that report beneficial metabolic effects from moving (without purposeful physical exercise) compared to sitting, including less body fat gain. While sitting at a desk, energy expenditure is just 5% above the basal level, whereas the value at least doubles within minutes of standing and walking [54].

### Housing environment and sleep duration or quality

Epidemiological studies concur in an association between increased exposure to residential traffic, noise, and fine airborne particulate matter and a higher risk of T2D diagnosis during the following 5–12 years. The risk was higher by roughly 20–40% for persons exposed to an, at least, 10 dB higher noise level or to 10 μg/m^3^ more of fine particulate matter over 10 years, or living on a busy road. It cannot be excluded that this association is not causal, but extensive adjustments have been made for age, sex and lifestyle (including BMI and physical activity), as well as for socioeconomic status, without loss of the observed associations [55–59].

Contributing factors are the duration and quality of sleep [60]. Night-time exposure to noise or light may cause sleep disturbances [61]. Similar effects have been reported for shift-workers or for persons with decreased sleep duration due to extended working hours or leisure time activities [62]. A recent meta-analysis of prospective studies reported the lowest risk of diabetes for 7–8 hours per day of sleep and an increased risk by 9% for each 1-h shorter sleep duration [63]. Longer sleep duration or day time napping may also be a risk factor for later diabetes or metabolic syndrome, but findings are not consistent [63–66]. In controlled trials performed in sleep laboratories, sleep restriction for 5 days caused a 29% decrease of whole body insulin sensitivity [67], and a decreased glucose disposal rate was observed already after one night of 4 hours of sleep [68].

It is conceivable that other aspects of the housing environment may also modulate diabetes risk, such as the climate, UV or ionising radiation, or exposure to toxins or allergens, but this area is not well researched.

### Coffee, tea, alcohol and smoking

A recent meta-analysis of coffee consumption confirmed an inverse dose-response relationship between caffeinated or decaffeinated coffee intake and risk of T2D, with a 25–30% lower risk for drinking three or more cups per day [69]. It still is not clear whether this reflects a causal relationship since controlled short-term trials only reported small changes in insulin and glucose responses to a glucose load after coffee consumption, varying from some improvement to modest impairment [70–73]. However, other inflammatory risk markers of T2D may be modified [74, 75]. In controlled trials, tea, in particular flavanol-rich green tea, has been reported to exert a modest improvement in glycaemic control if more than three cups or the equivalent amount of green tea catechins were consumed. A meta-analysis of 22 trials reported a mean decrease of fasting blood glucose by 1.4 mg/dL [76]. Epidemiological studies suggest a modest decrease of T2D risk by 10–15% in those drinking more than three cups per day [77, 78].

The health risks of alcohol intake seem to be dose dependent. There is now consistent epidemiological data that moderate alcohol consumption (1–2 drinks per day) may reduce the risk of developing T2D by a mean maximum of approximately 20%, but possibly only in women and not in Asian populations [79, 80]. A well-controlled study of the consumption of 150 mL of wine for dinner in patients with T2D observed a modest improvement of cardiometabolic parameters after 2 years [81].

Conversely, exposure to cigarette smoke both passively and actively has been found to be associated with increased risk of T2D when compared to non-smokers [82]. Meta-analyses of prospective cohort studies reported a considerably higher relative risk of diabetes for heavy smokers (risk ~1.6) than for lighter smokers (risk ~1.3) or for former smokers (risk ~1.2) [83, 84]. Interestingly, a recent study reported no association between smoking and incident T2D in a large multi-ethnic cohort, which suggests a more complex role of smoking in causing diabetes [85].

### Depression and stress as risk factors

Stress at work, in social relationships or in other aspects of life is difficult to define given that it is the impact on the individual and the coping mechanisms that are probably relevant, i.e. perceived stress. Thus, the results of cross-sectional or prospective studies on the association of stress with T2D have been variable [86–90]. However, a 35-year study of perceived stress in Swedish men reported a significant association with later diabetes, and a similar result was observed in persons with burn-out syndrome [91, 92]. More consistent is the observation of an increased diabetes risk in persons with symptoms of depression or anxiety, and there appears to be a bidirectional relationship between depressive mood and diabetes [89, 93–97]. Interestingly, living alone is associated with an increased risk of T2D in men (hazard ratio 1.89), but not in women [98].

## Impact of socioeconomic status

An inverse association of T2D and socioeconomic position has been reported worldwide, also after separate analysis of high-, middle- and low-income countries, independent of whether measured by educational level, occupation or income [99, 100]. Low levels of socioeconomic determinants were associated with a 40–60% higher relative risk compared to the subgroup with high levels. In the English Longitudinal Study of Ageing, the lowest life course socioeconomic status group experienced a more than doubled risk of diabetes [101]. An analysis in Europe found most of the difference to be mediated by BMI [102]. A study of health behaviours in Australia found smoking and lack of physical activity as a major mediator of the increased diabetes incidence in persons with low socioeconomic status [103]. A tentative conclusion is that an increased income may lower the risk of T2D if accompanied by an appropriate change in diet and lifestyle.

## Infections as a cause of T2D?

Is there any indication for an infectious origin of T2D? A non-diabetic partner of a diabetic spouse has a 26% increased diabetes risk [104], likely due to a ‘contagious’ lifestyle. Additionally, adenovirus subtype 36 infections have been closely associated with obesity in several regions of the world, and a causal relationship was established in animal experiments [105, 106]. However, adenovirus-36 antibodies are uncommon in T2D and are associated with increased rather than decreased insulin sensitivity [107]. Nevertheless, T2D has been clearly associated to certain infections, such as hepatitis C virus, which may lead to hepatic steatosis, insulin resistance, T2D and cardiovascular disease [108, 109], or *Chlamydia pneumoniae*, which may cause β-cell dysfunction in the context of systemic inflammation [110]. The diabetes promoting effects of antiretroviral therapy should also be mentioned here [111].

These findings argue against the presence of a specific infectious agent in the aetiology of T2D but leave room for a role of chronic infections and associated systemic inflammation in promoting insulin resistance.

## How may unfavourable lifestyle and environmental changes cause the current T2D epidemic?

Under favourable lifestyle and environmental conditions, people who exhibit a high genetic or epigenetic risk are at increased risk of T2D. Diabetes risk genes seem to directly or indirectly (via insulin resistance) affect β-cell function [112]. Pima Indians are people with a strong genetic predisposition for T2D and they progress to T2D even when living a ‘normal’ lifestyle in a ‘normal’ environment [113]. With an unfavourable change in lifestyle and environment between 1995 and 2010, non-Pima neighbours also began to exhibit an increased diabetes rate [113].

It seems unlikely that the same type of changes in lifestyle and environment can be held responsible in countries with widely different socioeconomic, cultural, environmental and lifestyle conditions. However, it appears evident that the diverse changes in lifestyle and environment have led to an increased prevalence of the primary diabetes risk factor (aside from age), namely a rise in the mean BMI in populations worldwide [3]. Even if overall obesity seems to be levelling off, such as in a region of China [114], increases in abdominal obesity are still rising.

In a 13-year observational study in the USA [115], an initial modestly elevated BMI of 27, when compared to an initial BMI of 22, resulted in an approximately three-fold increase of diabetes risk. Therefore, the global obesity epidemic probably translates some of the changes in lifestyle and environment into a higher T2D risk. However, in the UK, a prospective study from 1984 to 2007 found that BMI could explain only 26% of the T2D increase [116], leaving room for lifestyle factors with little impact on body weight such as lack of physical activity.

Epidemiological analyses suggest that many of the diabetes risk factors described above contribute at least in part or independently to disease development, such as amount and type of food, sedentary time, physical activity, watching TV, noise, fine dust, sleep duration, shift working, emotional stress, socioeconomic status and some infections (for references see above). The various risk factors are not expected to directly interact with the same target in the human organism. However, since the loss of insulin production is the ultimate cause of developing overt T2D, environmental and lifestyle factors must directly or indirectly cause β-cell damage. Concomitant morphological changes in pancreatic islets, either because of β-cell death or because of dedifferentiation, have indeed been observed in the pathogenesis of T2D [117].

**Fig. 1:**
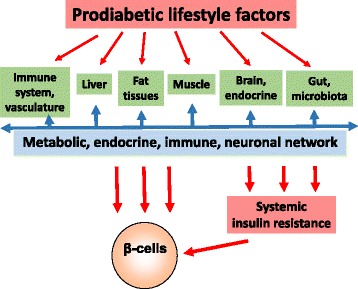
Prodiabetic lifestyle factors target regulatory networks. Although the widely different lifestyle-defined diabetes risk factors are expected to interact with different parts of the body, they probably target the metabolic, endocrine, immune and neurological network(s) and synergise in promoting β-cell damage

Only few environmental or lifestyle factors are expected to directly affect β-cell function, possible exceptions are high levels of nutrients or their metabolites in blood as one cause of metabolic stress [118–120]. Other diabetes risk factors may not directly target β-cells but have distant sites of action, such as the immune system (immune mediators), vasculature (e.g. immune mediators, adhesion molecules), fat tissue (adipokines), liver (glucose, lipids, fetuin A, immune mediators), muscle (myokines), brain (neurohormones and signals), the intestine (incretins), or microbiota (short-chain fatty acids, lipopolysaccharides). Because of the crosstalk between these organs, it is difficult to disentangle metabolic from endocrine, immunological or neuronal mechanisms of diabetes risk factors (Fig. 1). For instance, all diabetes risk factors discussed above have been reported to promote an inflammatory state and concomitant insulin resistance [121–124]. Conversely, diabetes-protective factors appear to exhibit anti-inflammatory activity [125]. Exposure to road traffic or fine dust is associated with increased serum levels of C-reactive protein or white blood cells [126, 127]. Sleep deprivation is associated with increased inflammatory reactivity, even after just one night of sleep loss [128, 129]. A western diet with refined sugars/starch and saturated fat is known to promote acute (lasting 1–4 hours) upregulation of pro-inflammatory immune mediators [130, 131], and there is sustained low-grade systemic inflammation in the context of an increase in BMI or visceral fat mass [132, 133]. The microbiota composition may promote systemic inflammation by causing gut leakage and release of lipopolysaccharides into the circulation [134, 135]. Persons with a sedentary lifestyle exhibit higher concentrations of circulating pro-inflammatory mediators [136–138]. Physical activity is known to attenuate low-grade inflammation [139, 140]. Fetuin released from the liver targets the same receptor as lipopolysaccharide [141]. Depression is also closely associated with elevated levels of inflammatory mediators and, conversely, subclinical inflammation may promote the occurrence of depressive symptoms [142–144].

Neuronal activity in response to environmental conditions may also affect β-cell function. The mechanisms involved include activation of the sympathetic nervous system and the secretion of catecholamines. Further, the hypothalamus–pituitary–adrenal axis may be activated, resulting in increased systemic levels of cortisol [145–147]. The development of food addiction also translates a non-healthy lifestyle into increased diabetes risk [148].

Genome-wide association and Mendelian randomisation studies of single nucleotide polymorphisms currently do not allow the definition of a causal role of metabolic versus endocrine, immunological or neuronal mediators of environmental/lifestyle effects in diabetes development. This may be due to the fact that these genetic polymorphisms explain only approximately 15% of T2D heritability [149, 150].

## Lifestyle changes for diabetes prevention

Trials of diabetes prevention have appreciated the contribution of lifestyle to diabetes risk. A primary strategy of such trials has been the targeting of moderate weight loss via lifestyle changes, including dietary measures and increased physical activity. After 3–4 years of such a lifestyle program in persons at high risk of T2D, there was a decrease in incident diabetes by 58% when compared to the control group in both landmark trials, namely the Diabetes Prevention Program and the Diabetes Prevention Study [151, 152]. In both trials, body weight loss during the first 2 years was only approximately 5%. Nevertheless, weight reduction in obese people by just 5–10% preferentially decreased the amount of visceral fat [153, 154], suggesting that this is a major target of the lifestyle intervention assessed. In obese people with T2D of short duration, a very low calorie diet caused normalisation of glycaemia within 1 week, with a concomitant reduction in hepatic triacylglycerol content by 30%, whereas body weight decreased by only 3% [155]. Further, there was substantial recovery of β-cell function after 8 weeks of a low calorie diet. A protein-rich, low calorie formula diet for meal replacement was found to substantially improve metabolic control, decrease the amount of antidiabetic medication and lower body weight in persons with insulin-treated T2D [156].

Long-term data are available from the two early diabetes prevention trials. After a mean follow-up of 15 or 9 years, respectively, the incidence of diabetes was 60% in the control group versus 52% in the intervention group in the Diabetes Prevention Program, and 56% versus 41% in the Diabetes Prevention Study [157, 158]. Thus, the lifestyle changes in the two studies halted the progression towards overt T2D in a large fraction of study participants for a few years, but was much less effective during follow-up. In both studies, there was a substantial regain of body weight over time. It is probable that long-term maintenance of body weight reduction would have prevented progression to T2D also in the long term, since this correlation is observed in people at risk of T2D after bariatric surgery [159].

## Conclusions

A host of environmental or lifestyle-dependent T2D risk factors have been described in prospective epidemiological studies, ranging from energy-dense food consumption to long-term exposure to high levels of fine dust. In these studies, the amount of personal, environmental or lifestyle aspects data that can or has been documented is limited, leaving room for confounding. For instance, the number of meals prepared at home or the frequency of tooth-brushing are rarely documented although both impact diabetes risk [160, 161] and may therefore confound associations with other lifestyle factors. Further, which environmental or lifestyle facotrs are associated with diabetes risk but do not bear a cause-effect relationship remains unknown.

Combining epidemiological data with the experience from intervention trials may allow to tentatively define diabetes protective factors. A consistent finding from prospective studies is the association of plant food-based diets with a lower T2D risk [14–16, 18, 20]. This fits with the outcome of the PREDIMED trial of Mediterranean diet, suggesting that the diabetes-protective effect of body weight reduction can be replaced, at least in part, by an appropriate quality diet [32]. Prebiotic plant fibres promote growth of a diverse and apparently healthy microbiota with less endotoxin leakage [162–164]. This may avoid inflammatory activation of endothelial and Kupffer cells in the liver with concomitant hepatocyte dysfunction, as usually observed in response to a western-type high-fat diet or during non-alcoholic fatty liver disease [165, 166]. A fibre-rich diet gives rise to the enhanced production of short-chain fatty acids by gut bacteria [167]. There is direct binding of these products to the free fatty acid receptor 2 on β-cells and the promotion of cell growth and function [168].

A second major diabetes-protective component of plant food are phytochemicals. A property common to most phytochemicals is the activation of cell defence and anti-inflammatory genes, for instance, via the Nrf2 signalling pathway [169]. These effects occur body-wide and are also demonstrable in β-cells [170].

A third major diabetes-protective factor is exercise. Interestingly, muscle work also activates cell defence and anti-inflammatory pathways via Nrf2 signalling. There is a direct beneficial effect of exercise on β-cell function [171], and also on liver function in non-alcoholic fatty liver disease, although there is no body weight loss [172].

The strongest diabetes-protective factor appears to be the avoidance or correction of a high body fat mass, notably visceral and ectopic fat. There is rapid improvement, and often normalisation, of glucose homeostasis within the first week after bariatric surgery or initiation of a very low calorie diet [155, 173]. After Roux-en-Y gastric bypass several rapid changes have been noted aside from the lower insulin and plasma glucose levels, including lower blood levels of leptin and branched amino acids, higher concentrations of adiponectin and bile acids, and stronger post prandial increases of GLP-1 and of the satiety peptide YY [173]. However, many of these changes are not seen after gastric banding or a very low calorie diet, such as no increased production of GLP-1 [155, 174, 175], bile acids [176, 177], peptide YY [178], or no decreased levels of branched amino acids [179]. Therefore, it seems probable that the major determinant of rapid metabolic improvement seen with Roux-en-Y gastric bypass, gastric banding or a very low calorie diet is the acute negative energy balance, which is similar for the three procedures [180, 181].

Obviously, the immediate response to severely restricted calorie uptake in the days after surgery or during a very low calorie diet is substantially less post-prandial insulin production, thus alleviating stress from β-cells. The lower level of protein synthesis decreases the demand of ATP from mitochondria, thereby reducing the amount of concomitantly released radical oxygen species and allowing recovery of mitochondrial proteins from oxidative damage such as nitrosylation or carbonylation [182, 183]. Indeed, within 1 week there is partial recovery of insulin production after the introduction of a very low calorie diet, with further normalisation in the following months [155, 184]. Recovery of β-cell function to varying extents is also seen early after gastric bypass [185–187].

A second immediate response to a very low calorie intake is the adaptation of metabolic control to the low amount of digestible carbohydrate. Such adaptation is primarily important in the liver, where most of gluconeogenesis takes place, including the synthesis of glucose from glycerol of triglycerides and metabolic breakdown of released fatty acids to the acetate level. Excess acetyl-CoA gives rise to acetoacetyl-CoA and, subsequently, to acetone and ß-hydroxybutyrate. The latter (reduced) ketone can be used as a substrate for oxidative energy production in place of glucose in peripheral tissues and can pass the blood–brain barrier [188].

It has been proposed that calorie restriction mimetics may elicit similar cellular responses as seen during an acute negative balance, including an improved cellular resistance to oxidative stress and an anti-inflammatory milieu [189, 190]. Many phytochemicals have been shown to exhibit activities related to calorie restriction, including the grape polyphenol resveratrol [191], anacardic acid from cashews, curcumin from the spice turmeric, garcinol from the fruit of the Kokum tree, epigallocatechin-3-gallate from green tea, and spermidine from fermented soy beans or wheat germs [192]. Therefore, it is not surprising that plant-based diets have been recognised to partly mimic the effects of calorie restriction [193, 194].

Taken together, intervention trials of diabetes prevention indicate that long-term preservation of normoglycaemia in people at high risk of T2D is achievable. Further research on the protective mechanisms associated with physical activity, healthy eating patterns and specific food components, anti-inflammatory strategies, or with weight reduction via low calorie diet or bariatric surgery seems warranted (Box 2).

## Box 1. Lifestyle characteristics conferring risk^a^ for type 2 diabetes as suggested by epidemiological studies

Diets poor in fibre, phytochemicals or plant food in general (relative risk increase by 44% to three-fold [14, 15])

Regular consumption of sugar-sweetened beverages (relative risk increase by 20–30% compared to non-consumption [22–24])

Little physical activity (leisure time/occupational) (relative risk approximately 40% higher compared to high total physical activity [41, 42])

Prolonged TV and monitor viewing/sedentary time (relative risk increased by approximately 3% per hour television watching [49])

Exposure to road traffic (noise, fine particulate matter) (relative risk increased by 20–40% for exposure to 10 dB higher noise level or 10 μg/m^3^ more of fine dust particles [55–59])

Smoking (relative risk increased by approximately 30%/60% for light/heavy smokers [85])

Short sleep duration and poor quality (relative risk increased by approximately 9% for every hour of shorter sleep duration [63])

Low mood/stress/depression (relative risk increase highly variable, depending on definition of stress and depression)

Low socioeconomic position (relative risk increased by 40–100% when compared to high socioeconomic level [99–101])

Infection with hepatitis C virus or *Chlamydia pneumoniae* (no epidemiological data on relative risk increase available)

^a^All factors remain significantly associated after statistical adjustment for body mass index (BMI) and other confounders, as described in the text. Virtually all non-infectious pro-diabetic lifestyle characteristics promote an increase in BMI and waist circumference.

## Box 2 Key points

- The global type 2 diabetes (T2D) epidemic is generally believed to result from environmental and lifestyle changes
- Diabetes risk factors include energy-dense western style diets, decreased physical activity, increased sitting and monitor viewing time, exposure to noise or fine dust, short or disturbed sleep, smoking, stress, depression, and a low socioeconomic status
- It is suggested that the various diabetes risk factors probably target different organs of the body, but these are connected by endocrine, metabolic, immune and neurological networks
- Since the loss of insulin production is the ultimate cause of developing overt T2D, environmental and lifestyle factors must directly or indirectly cause β-cell damage
- Epidemiological studies indicate that major diabetes-protective factors comprise plant food-based diets and moderate to high intensity muscle work
- It may be possible to reproduce, at least in part, the benefits of body weight reduction by calorie restriction mimetics
